# Hypomyelinating Leukodystrophy 8 (HLD8)-Associated Mutation of POLR3B Leads to Defective Oligodendroglial Morphological Differentiation Whose Effect Is Reversed by Ibuprofen

**DOI:** 10.3390/neurolint14010018

**Published:** 2022-02-16

**Authors:** Sui Sawaguchi, Rimi Suzuki, Hiroaki Oizumi, Katsuya Ohbuchi, Kazushige Mizoguchi, Masahiro Yamamoto, Yuki Miyamoto, Junji Yamauchi

**Affiliations:** 1 Laboratory of Molecular Neurology, Tokyo University of Pharmacy and Life Sciences, Hachioji 192-0392, Japan; s167023@toyaku.ac.jp (S.S.); kasumisou.r@docomo.ne.jp (R.S.); miyamoto-y@ncchd.go.jp (Y.M.); 2 Tsumura Research Laboratories, Tsumura & Co., Inashiki 200-1192, Japan; ooizumi_hiroaki@mail.tsumura.co.jp (H.O.); oobuchi_katsuya@mail.tsumura.co.jp (K.O.); mizoguchi_kazushige@mail.tsumura.co.jp (K.M.); hirokoma@h.email.ne.jp (M.Y.); 3 Department of Pharmacology, National Research Institute for Child Health and Development, Setagaya 157-8535, Japan

**Keywords:** Pelizaeus–Merzbacher disease (PMD), hypomyelinating leukodystrophy (HLD), POLR3B, lysosome, oligodendrocyte, ibuprofen

## Abstract

POLR3B and POLR3A are the major subunits of RNA polymerase III, which synthesizes non-coding RNAs such as tRNAs and rRNAs. Nucleotide mutations of the RNA polymerase 3 subunit b (polr3b) gene are responsible for hypomyelinating leukodystrophy 8 (HLD8), which is an autosomal recessive oligodendroglial cell disease. Despite the important association between POLR3B mutation and HLD8, it remains unclear how mutated POLR3B proteins cause oligodendroglial cell abnormalities. Herein, we show that a severe HLD8-associated nonsense mutation (Arg550-to-Ter (R550X)) primarily localizes POLR3B proteins as protein aggregates into lysosomes in the FBD-102b cell line as an oligodendroglial precursor cell model. Conversely, wild type POLR3B proteins were not localized in lysosomes. Additionally, the expression of proteins with the R550X mutation in cells decreased lysosome-related signaling through the mechanistic target of rapamycin (mTOR). Cells harboring the mutant constructs did not exhibit oligodendroglial cell differentiated phenotypes, which have widespread membranes that extend from their cell body. However, cells harboring the wild type constructs exhibited differentiated phenotypes. Ibuprofen, which is a non-steroidal anti-inflammatory drug (NSAID), improved the defects in their differentiation phenotypes and signaling through mTOR. These results indicate that the HLD8-associated POLR3B proteins with the R550X mutation are localized in lysosomes, decrease mTOR signaling, and inhibit oligodendroglial cell morphological differentiation, and ibuprofen improves these cellular pathological effects. These findings may reveal some of the molecular and cellular pathological mechanisms underlying HLD8 and their amelioration.

## 1. Introduction

POLR3A and POLR3B are important subunits of RNA polymerase III, which is a synthesizing non-coding RNA that includes a variety of tRNAs and rRNAs. Nucleotide mutations of the RNA polymerase 3 subunit b (polr3b) gene are responsible for autosomal recessive hypomyelinating leukodystrophy 8 (HLD8) [1,2,3,4]. HLD8 is a typical oligodendroglial cell genetic disease [1,2,3,4]. Despite these detailed studies on the relationship between genes encoding POLR3A or POLR3B and disease pathologic features, therapeutic drugs and strategies for HLD8 have not been established. Hypomyelinating leukodystrophies (HLDs) are a recently classified group of hereditary neuropathies [5,6,7,8]. These diseases are rare, affecting one out of every 250,000 to 500,000 people [5,6,7,8]. Oligodendrocytes (also called oligodendroglial cells) play a role in generating the myelin sheath [9,10,11,12]. They functionally contribute to the propagation of saltatory conduction, and they protect neuronal axons from a variety of stresses such as physical and physiological stresses [9,10,11,12].

Because the HLD8-associated mutation of POLR3B decreases the POLR3B protein expression level, it is thought to be a loss-of-function mutation. However, some residual proteins with the HLD8-associated mutation seem likely to remain in pathological cells and tissues [2,3,4]. Therefore, these residual proteins may cause molecular and cellular pathological effects of HLD8. Herein, we report that a severe HLD8-associated nonsense mutation of Arg550-to-Ter (R550X) localizes POLR3B proteins as aggregates, which is similar to other HLD-associated mutated proteins [13,14,15,16,17,18] in FBD-102b cell lysosomes, and it is used as an oligodendroglial precursor cell model. However, the wild type proteins were not localized in lysosomes. Additionally, in cells expressing proteins with the R550X mutation, lysosome-related signaling of the mechanistic target of rapamycin (mTOR) such as phosphorylation of ribosomal S6 and 4E-BP1 proteins [19,20,21,22,23,24] was greatly decreased. Additionally, while cells harboring the R550X mutant constructs exhibited decreased morphological differentiation, cells harboring wild type constructs showed differentiated phenotypes. Furthermore, molecular and cellular phenotypes harboring the mutant constructs were improved by ibuprofen, which is a non-steroidal anti-inflammatory drug (NSAID) that has been reported to have protective effects preferentially in neuronal cells but not in glial cells [25,26].

Thus, the HLD8-associated mutation of POLR3B proteins affects oligodendroglial cell morphological differentiation possibly through decreased lysosome-related mTOR signaling, and treatment with ibuprofen can cause its amelioration in glial cells such as oligodendroglial cells. This suggests some underlying molecular and cellular pathological mechanisms for HLD8.

## 2. Material and Methods

### 2.1. Plasmid Constructions and Primary and Secondary Antibodies

The full-length human polr3b (Gene ID 55703) gene, which is inserted into a vector expressing GFP, was purchased from Sino Biological, Inc. (Beijing, China). The polr3b cDNA harboring the Arg550-to-Ter (R550X) mutation (OMIM ID 614366) was generated with a Gflex DNA polymerase (Takara Bio, Shiga, Japan)-based method using human polr3b gene as template. DNA sequences were confirmed by Fasmac sequencing service (Kanagawa, Japan). Antibodies that were used in these experiments are listed in Table 1.

### 2.2. Cell Culture

The FBD-102b cell line (a mouse brain oligodendrocyte precursor cell line) was kindly provided by Dr. Y. Tomo-oka (Tokyo University of Science, Chiba, Japan; Riken, Saitama, Japan). FBD-102b cells on cell culture dishes (Greiner, Oberösterreich, Germany) or glass coverslips (Matsunami Glass, Osaka, Japan) were cultured in DMEM/F-12 (Nacalai Tesque, Kyoto, Japan) containing 10% heat-inactivated FBS (Thermo Fisher Scientific, Waltham, MA, USA) and PenStrep (Thermo Fisher Scientific) in the presence or absence of ibuprofen (300 micromolar, Nacalai Tesque) [25,26]) in 5% CO_2_ at 37 °C.

### 2.3. Cell Differentiation

FBD-102b cells were cultured in medium without FBS on the cell culture dishes using advanced TC polymer modification (Greiner) in 5% CO_2_ at 37 °C to induce differentiation. After a few days, cells grow to have a widespread membrane that is similar to the myelin membrane. Their cellular morphological phenotypes were identified as differentiated cells [27].

We confirmed that FBD-102b cells were viable under each differentiation experimental condition by verifying that attached trypan-blue (Nacalai Tesque)-incorporating cells comprised less than 5% of all cells in each culture.

### 2.4. Cell Transfection

Cells were transfected with the respective plasmids using a Screen Fect A or Screen Fect A Plus transfection kit (Fujifilm, Tokyo, Japan), in accordance with the manufacturer’s instructions. The medium was replaced 4 h after transfection. Transfected cells were generally used for experiments 48 h after transfection. Otherwise, G418 (2500 microgram/mL)-resistant clones were collected as stable clones.

We confirmed that FBD-102b cells were viable under each transfection experimental condition by verifying that attached trypan-blue-incorporating cells made up less than 5% of all cells in each culture.

### 2.5. Confocal Microscopic Images

Cells on glass coverslips were fixed with 4% paraformaldehyde or 100% cold methanol and blocked with a Blocking One reagent (Nacalai Tesque). Then, cells were incubated with primary antibodies and secondary antibodies that were conjugated with Alexa Fluor dyes, in accordance with the manufacturer’s instructions. Each glass coverslip was placed on each slide glass and mounted with a Vectashield reagent (Vector Laboratories, Burlingame, CA, USA).

TIFF images of the cells were collected using a microscope equipped with a laser-scanning Fluoview apparatus (FV1000D or FV1200, Olympus, Tokyo, Japan) and processed using Fluoview software (ver. 2016, Olympus). The resulting color images were analyzed using Image J software (National Institutes of Health, Bethesda, MD, USA). Each image in each figure is representative of three independent experimental results.

### 2.6. Polyacrylamide Gel Electrophoresis and Immunoblotting

Cells were lysed in lysis buffer A (50 mM HEPES-NaOH, pH 7.5, 150 mM NaCl, 20 mM MgCl_2_, 1 mM phenylmethane sulfonyl fluoride, 1 microgram/mL leupeptin, 1 mM EDTA, 1 mM Na_3_VO_4_, 10 mM NaF, and 0.5% NP-40) [27,28,29,30]. After centrifugation, the supernatants were incubated with a non-denaturing sample buffer (also called a native polyacrylamide gel sample buffer; Nacalai Tesque) or a denaturing sample buffer (Nacalai Tesque) for non-denaturing and denaturing conditions, respectively. Then, their samples were separated on non-denaturing or denaturing polyacrylamide gels (Nacalai Tesque). The electrophoretically separated proteins were transferred onto polyvinylidene difluoride membranes (Merck-Millipore, Darmstadt, Germany) and blocked with Blocking One reagent, then immunoblotted with primary antibodies followed by secondary antibodies conjugated with HRP proteins. The bound antibodies were detected by X-ray film (Fujifilm) exposure using an Immuno Star Zeta reagent (Fujifilm).

Images were captured as TIFF files using LiDE scanners (Canon, Tokyo, Japan) and processed using LiDE driver software (ver. 2017, Canon). The band pixels were measured using Image J software (ver. 1.53). Each image in each figure is representative of three independent experimental results (Figure S1).

### 2.7. Statistical Analysis

Values are presented as the average ± standard deviation (SD) for separate experiments. Intergroup comparisons were made using the unpaired Student’s *t*-test and Excel software (ver. 2019, Microsoft, Redmond, WA, USA). A one-way analysis of variance (ANOVA) was followed by a Fisher’s protected least significant difference (PLSD) test as a post hoc comparison using StatPlus (add-in Exel Ver. 2019, AnalystSoft, Walnut, CA, USA). Differences were considered significant when *p* < 0.05.

### 2.8. Ethics Statement

Gene recombination techniques were performed in accordance with a protocol that was approved by the Tokyo University of Pharmacy and Life Sciences Gene and Animal Care Committee (Approved Nos. LS28-20 and LSR3-011).

## 3. Results

### 3.1. The R550X Mutant Proteins of POLR3B Are Aggregated in Lysosomes in FBD-102b Cells

**Figure 1:**
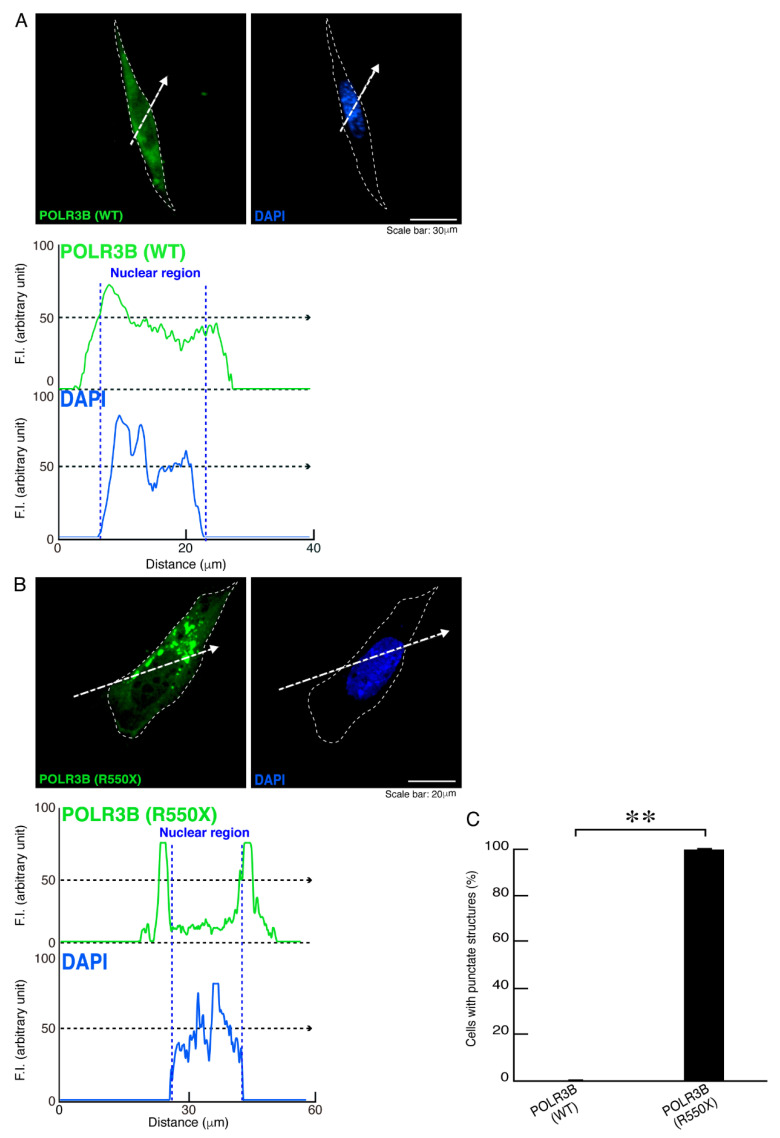
R550X mutant proteins of POLR3B accumulate in punctate structures in FBD-102b cells, whereas the wild type proteins are expressed throughout the cell body. (**A**,**B**) FBD-102b cells, surrounded by dotted lines, were transfected with the plasmid encoding the wild type (WT) POLR3B or the R550X mutant constructs. Transfected cells were detected using transfected proteins (green) and nuclear DAPI (blue). Scan plots were performed along the white dotted lines in the direction of the arrows in images. Graphs showing the fluorescence intensities (arbitrary units) along the white dotted lines in the direction of the arrows can be seen in the bottom panels. (**C**) Percentages of cells with punctate structures were statistically assessed (** *p* < 0.01; *n* = 3 fields).

Because R550X mutant proteins in POLR3B may be localized in punctate structures [13,14,15,16,17,18], we transfected the plasmid encoding the wild type or mutated POLR3B into FBD-102b cells. While the wild type POLR3B proteins were distributed throughout the cell body (Figure 1A), mutated POLR3B proteins were localized in punctate structures in the cytoplasmic regions in over 90% of the transfected cells (Figure 1B,C).

**Figure 2:**
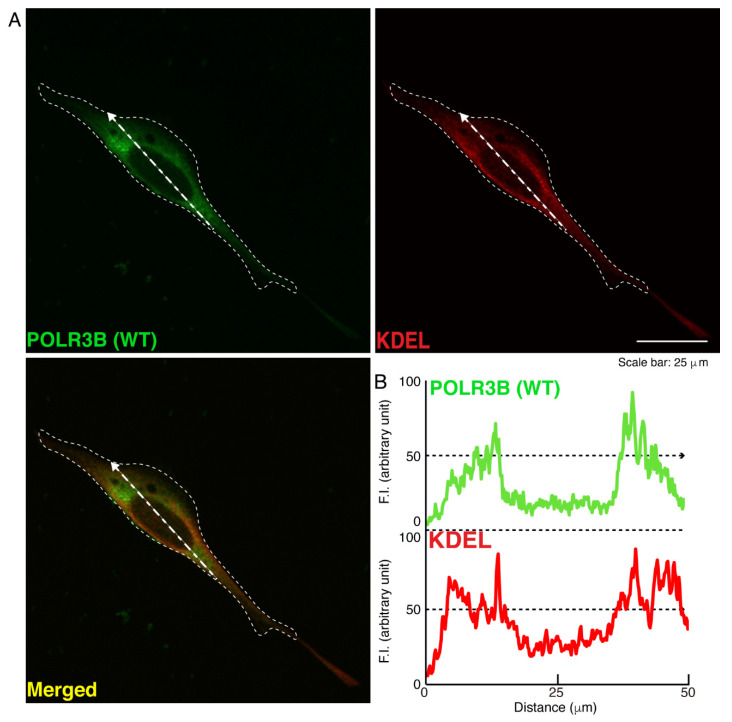
Wild type POLR3B proteins are not co-localized with the ER in cells. (**A**,**B**) Cells were transfected with the plasmid encoding the wild type (WT) POLR3B and detected with transfected proteins (green) and an antibody against the KDEL antigen (red). Scan plots were performed along the white dotted lines in the direction of the arrows in the color images (green and red as well as merged images). Graphs showing the fluorescence intensities (arbitrary units) along the white dotted lines in the direction of the arrows (black dotted lines in right bottom panels) can be seen in the right bottom panels.

**Figure 3:**
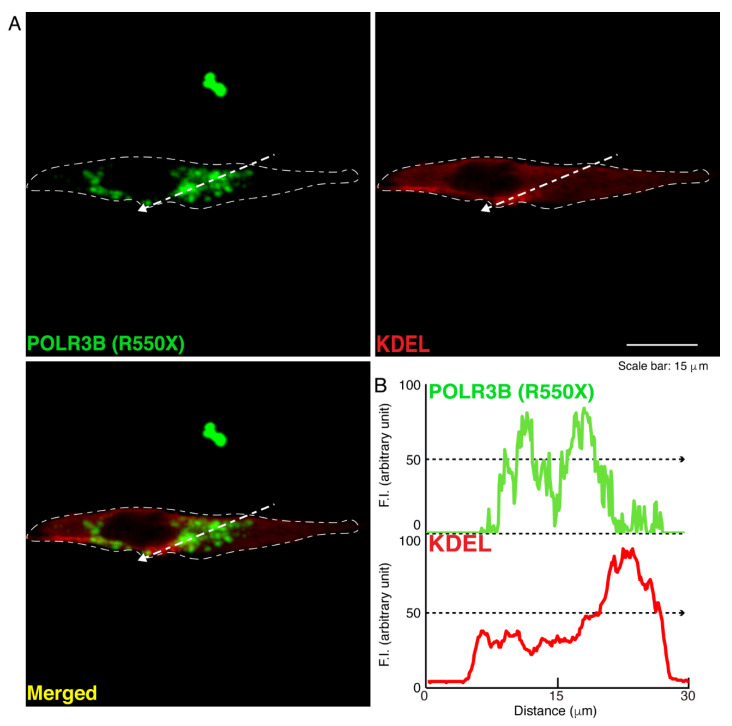
POLR3B mutated proteins are not co-localized with the ER in cells. (**A**,**B**) Cells were transfected with the plasmid encoding POLR3B (R550X) (green) and stained using an antibody against the KDEL antigen (red). Scan plots were performed along the white dotted lines in the direction of the arrows in the color images (green and red as well as merged images). Graphs showing the fluorescence intensities (arbitrary units) along the white dotted lines in the direction of the arrows (black dotted lines in right bottom panels) can be seen in the right bottom panels.

**Figure 4:**
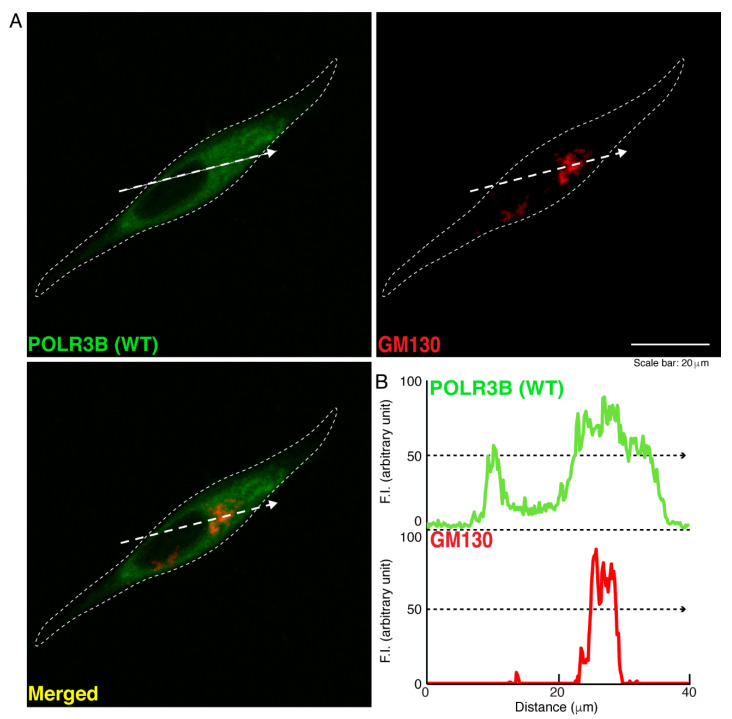
Wild type proteins are not co-localized with the Golgi body in cells. (**A**,**B**) Cells were transfected with the plasmid encoding the wild type (WT) POLR3B (green) and stained using an antibody against the GM130 antigen (red). Scan plots were performed along the white dotted lines in the direction of the arrows in the color images (green and red as well as merged images). Graphs showing the fluorescence intensities (arbitrary units) along the white dotted lines in the direction of the arrows (black dotted lines in right bottom panels) can be seen in the right bottom panels.

**Figure 5:**
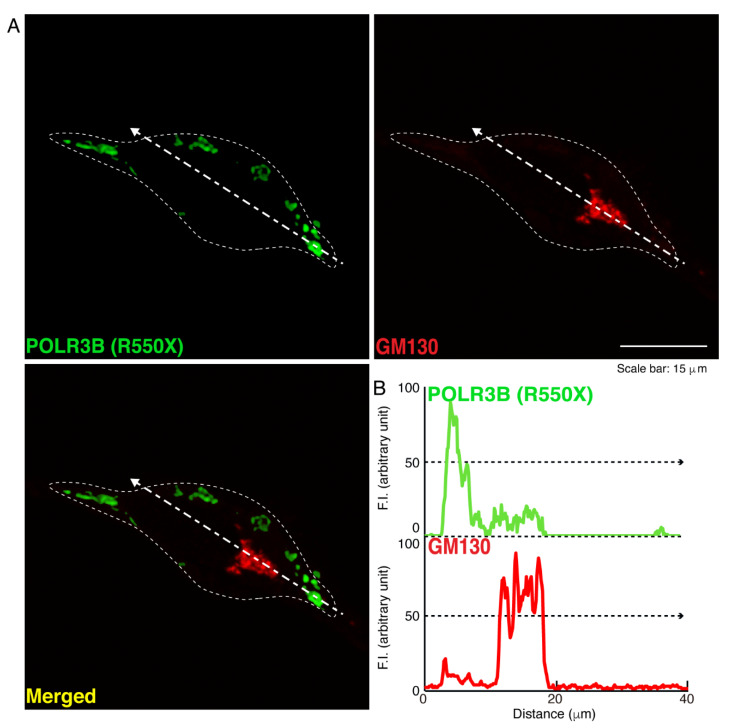
Mutated proteins are not co-localized with the Golgi body in cells. (**A**,**B**) Cells were transfected with the plasmid encoding POLR3B (R550X) (green) and stained using an antibody against the GM130 antigen (red). Scan plots were performed along the white dotted lines in the direction of the arrows in the color images (green and red as well as merged images). Graphs showing the fluorescence intensities (arbitrary units) along the white dotted lines in the direction of the arrows (black dotted lines in right bottom panels) can be seen in the right bottom panels.

**Figure 6:**
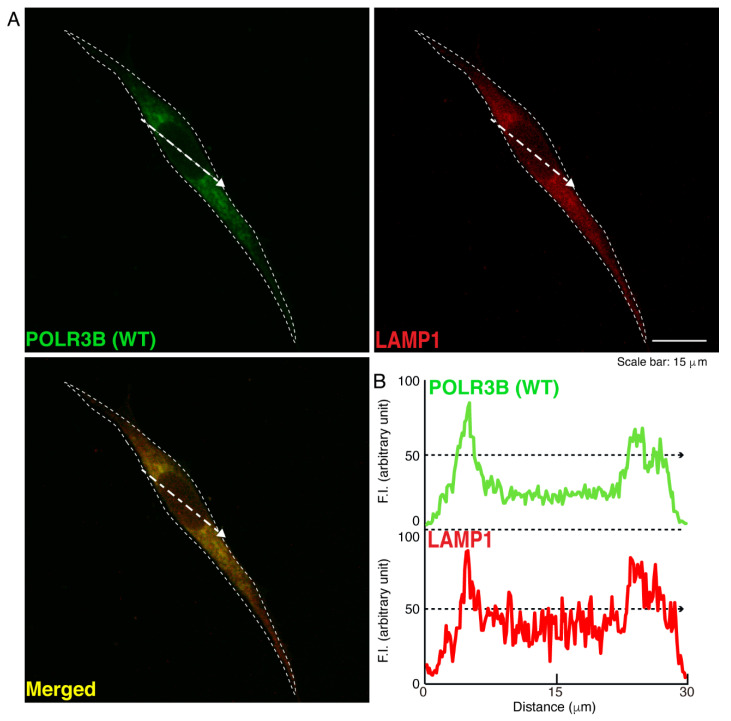
Wild type proteins are not significantly co-localized with the lysosome in cells. (**A**,**B**) Cells were transfected with the plasmid encoding the wild type (WT) POLR3B (green) and stained using an antibody against the LAMP1 antigen (red). Scan plots were performed along the white dotted lines in the direction of the arrows in the color images (green and red as well as merged images). Graphs showing the fluorescence intensities (arbitrary units) along the white dotted lines in the direction of the arrows (black dotted lines in right bottom panels) can be seen in the right bottom panels.

**Figure 7:**
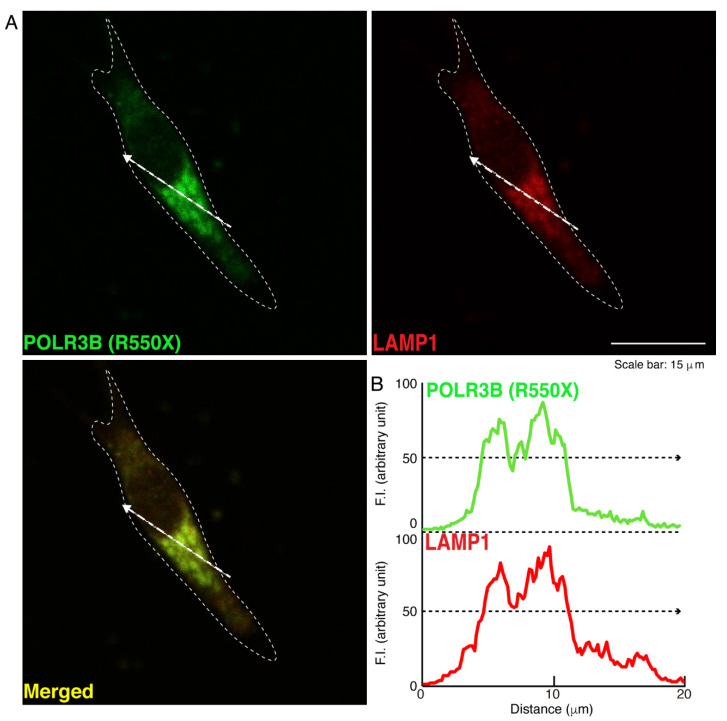
Mutated proteins are mostly co-localized with the lysosome in cells. (**A**,**B**) Cells were transfected with the plasmid encoding POLR3B (R550X) (green) and stained using an antibody against the LAMP1 antigen (red). Scan plots were performed along the white dotted lines in the direction of the arrows in the color images (green and red as well as merged images). Graphs showing the fluorescence intensities (arbitrary units) along the white dotted lines in the direction of the arrows (black dotted lines in right bottom panels) can be seen in the right bottom panels.

We then investigated which organelle corresponds to punctate structures. Wild type POLR3B proteins were not significantly co-localized with the endoplasmic reticulum (ER) antigen Lys-Asp-Asn-Leu (KDEL) (Figure 2). Additionally, mutated POLR3B proteins were not significantly co-localized with the KDEL antigen (Figure 3). However, because wild type POLR3B proteins were localized throughout cell bodies, wild type POLR3B proteins may be at least partially co-localized with major organelle antigens. Such localization for wild type POLR3B proteins was obtained for the Golgi body antigen Golgi matrix protein of 130 kDa (GM130) (Figure 4). Mutated POLR3B proteins were not co-localized with the GM130 antigen (Figure 5). Moreover, wild type POLR3B proteins throughout cell bodies exhibit a co-localization with the lysosome antigen lysosomal-associated membrane protein 1 (LAMP1) (Figure 6). Conversely, mutated POLR3B proteins were mostly co-localized with LAMP1, indicating that HLD8-associated mutation causes POLR3B proteins to localize in lysosomes (Figure 7). Additionally, although the wild type POLR3B proteins appeared to overlap with LAMP1 antigens, LAMP1 antigens in normal cellular states can be present throughout the cell body.

**Figure 8:**
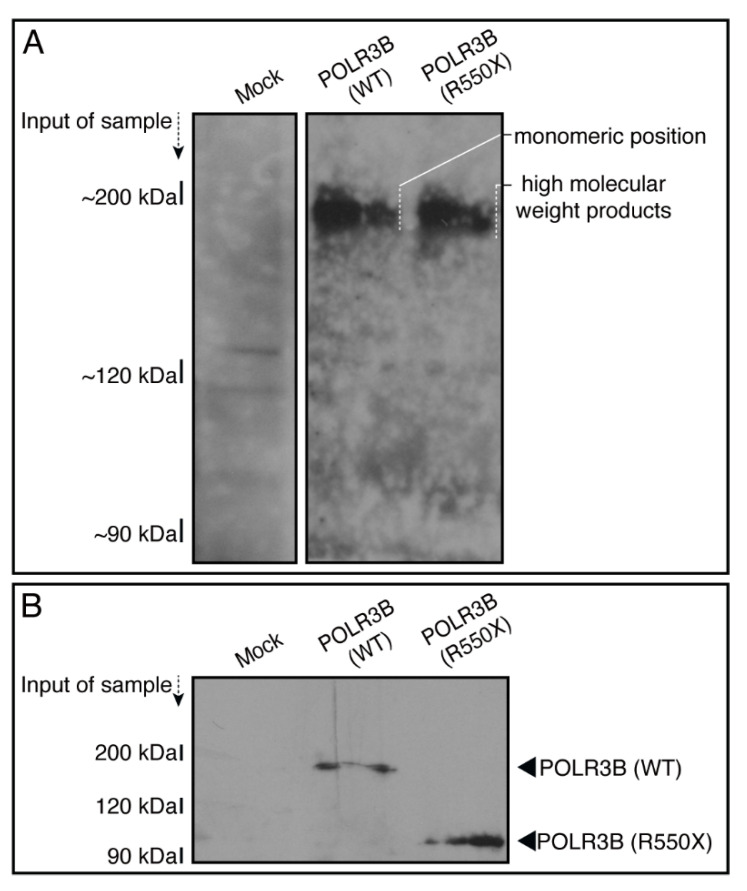
Mutated but not wild type proteins exhibit dimeric and high molecular weight structures in non-denaturing polyacrylamide gel electrophoresis. (**A**,**B**) The lysates of cells transfected with an empty vector or with a plasmid encoding the wild type (WT) POLR3B or POLR3B (R550X) were subjected to non-denaturing (upper images) and denaturing (lower images) polyacrylamide gel electrophoresis and detected using immunoblotting. The position corresponding to the molecular weight of the wild type monomer or the R550X monomeric or polymeric (including high molecular weight products) structures is shown.

We, thus, investigated whether mutated POLR3B proteins are aggregated and localized in lysosomes. The lysates of mock-transfected cells or cells expressing the wild type or mutated proteins were subjected to non-denaturing or denaturing polyacrylamide gel electrophoresis. In non-denaturing or denaturing polyacrylamide gel electrophoresis, the wild type proteins corresponded to the predicted molecular weight (~200 kDa), whereas mutated proteins displayed very high molecular weights (Figure 8A). In denaturing polyacrylamide gel electrophoresis, the mutated proteins corresponded to the predicted small molecular weight, indicating that HLD8-associated mutation leads to protein aggregation (Figure 8B).

### 3.2. Cells Expressing the R550X Mutant Proteins of POLR3B Fail to Exhibit Differentiation Phenotypes

**Figure 9:**
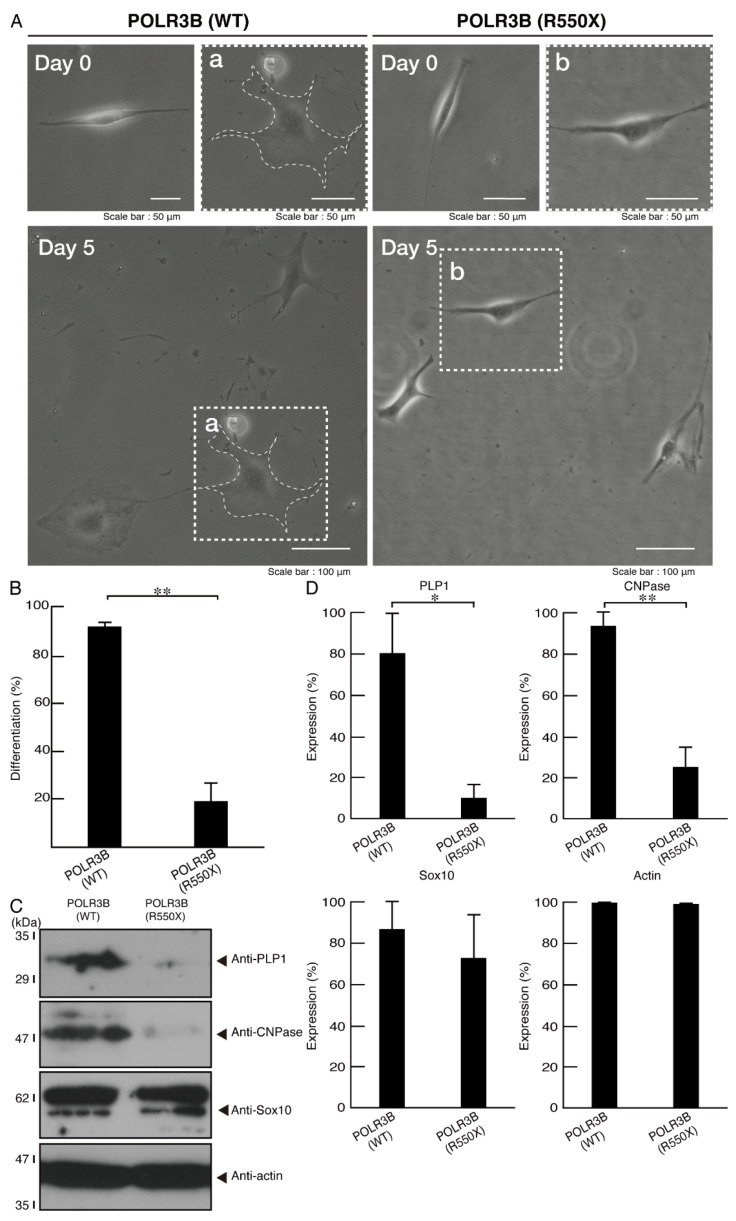
Cells harboring the mutants fail to undergo morphological differentiation. (**A**) Cells harboring the wild type (WT) or POLR3B (R550X) were allowed to differentiate for 5 days. Some cells in the bottom images are surrounded by white dotted lines (a and b). The square fields a and b indicated by the dotted lines in the bottom panels are magnified in the upper panels a and b. Images of cells at 0 day are also shown. (**B**) Cells with widespread membranes were statistically assessed (** *p* < 0.01; *n* = 5 fields (50 cells in total)). (**C**) The lysates of the respective cells were immunoblotted with an antibody against PLP1 and CNPase, cell lineage marker Sox10, and control actin. (**D**) Their expression levels are shown and statistically compared to their respective controls. (* *p* < 0.05 of Student’s *t*-test; *n* = 3 blots).

Because POLR3B mutated proteins have some molecular pathological properties, we further asked whether mutated proteins have effects on cell differentiation in FBD-102b cells. While cells harboring the wild type constructs exhibited differentiated phenotypes with widespread membranes following the induction of differentiation, cells harboring the R550X mutant constructs failed to show these differentiated phenotypes (Figure 9A,B). These results were supported by decreased expression levels of the differentiation and myelin marker proteins PLP1 and CNPase (Figure 9C,D). The oligodendrocyte-lineage marker Sox10 and the internal control protein actin were comparable in both cells, revealing that HLD8-associated mutation is related to an inhibition of morphological differentiation.

**Figure 10:**
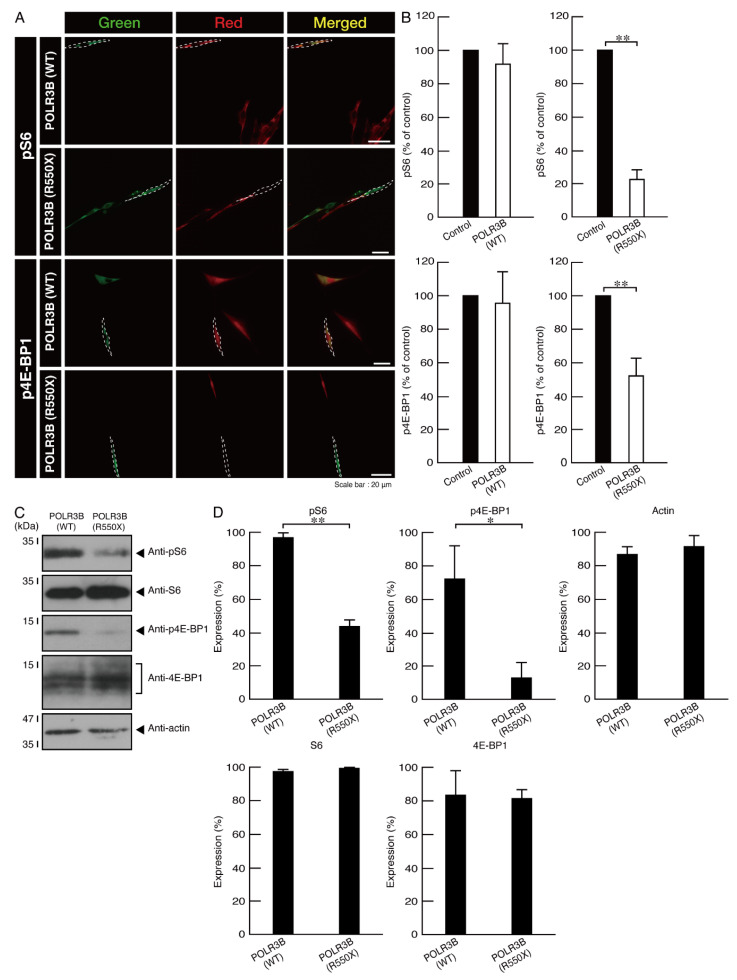
Cells harboring the mutants decrease phosphorylation levels of ribosomal S6 and 4E-BP1 proteins at the cell and protein levels. (**A**,**B**) Cells harboring the wild type (WT) or POLR3B (R550X) (green) were stained with an anti-(pS240 and pS244)ribosomal S6 protein (pS6) or anti-(pT37)4E-BP1 (p4E-BP1) antibody (red) that shows that phosphorylation acts downstream of mTOR signaling. Their phosphorylation levels are shown statistically compared to those in their respective control cells under each image (** *p* < 0.01; *n* = 3 fields). (**C**,**D**) The lysates of cells harboring the wild type (WT) or POLR3B (R550X) were immunoblotted with an anti-(pS240 and pS244)ribosomal S6 protein (pS6), anti-(pT37)4E-BP1 (p4E-BP1), anti-S6, and anti-4E-BP1. Statistically significant differences are shown in their immunoreactive bands (* *p* < 0.05 of Student’s *t*-test ** *p* < 0.01; *n* = 3 blots).

Because mutated proteins are localized as aggregates in lysosomes, we examined whether cells expressing the mutated proteins decrease signaling through mTOR. Signaling through mTOR is present around lysosomes that have activities that are strictly correlated with their signaling [19,20]. Additionally, mTOR signaling, including ribosomal S6 and 4E-BP1 protein phosphorylation, is required for oligodendroglial cell differentiation and subsequent myelination [21,22,23,24]. As expected, cells expressing the mutated, but not the wild type, proteins showed decreased ribosomal S6 protein phosphorylation (Figure 10A,B shows immunofluorescence; Figure 10C,D shows immunoblotting). Similar results were obtained for 4E-BP1 protein phosphorylation.

### 3.3. Ibuprofen Specifically Improves the Phenotypes of Cells Expressing the R550X Mutant Proteins of POLR3B

**Figure 11:**
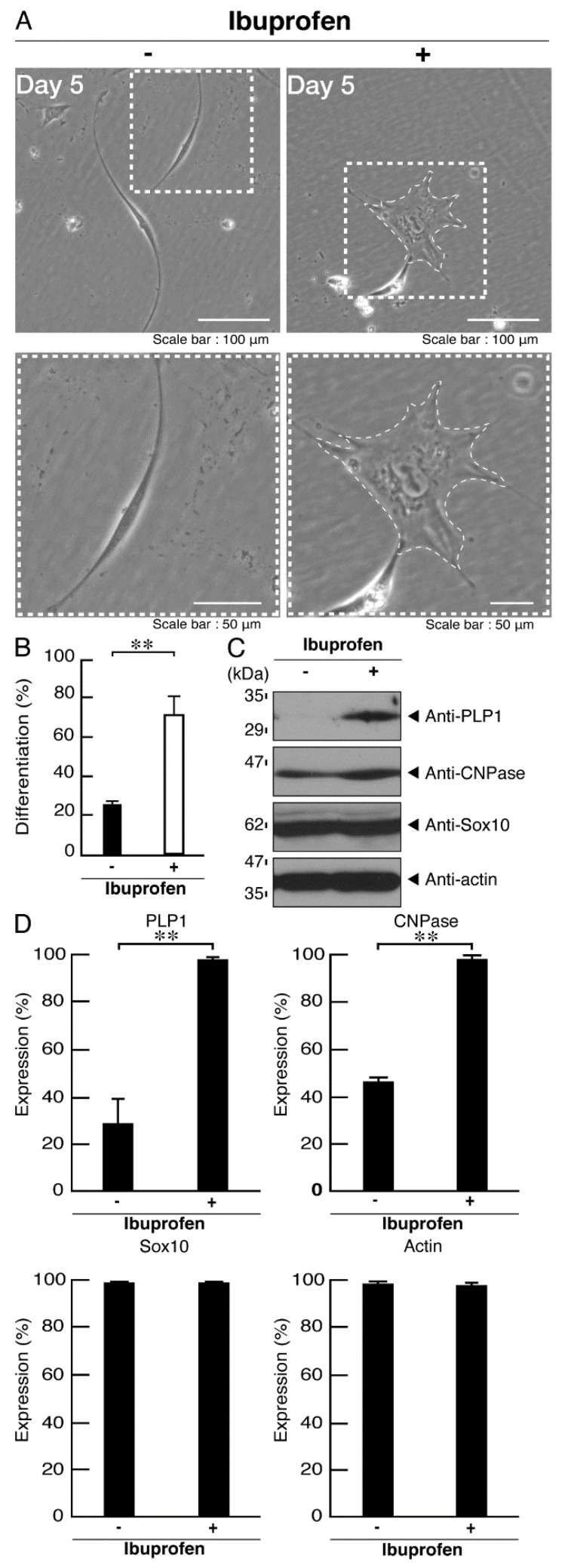
Ibuprofen improves phenotypes in cells harboring the mutants. (**A**) Cells harboring POLR3B (R550X) were allowed to differentiate in the presence or absence of ibuprofen for 5 days. Some cells in the upper images are surrounded by white dotted lines. The square fields indicated by the dotted lines in the upper panels are magnified in the respective lower panels. (**B**) Cells with widespread membranes were statistically assessed (** *p* < 0.01; *n* = 5 fields (50 cells in total)). (**C**) The lysates of the respective cells were immunoblotted with an antibody against PLP1, CNPase, Sox10, and actin. (**D**) Their expression levels are shown statistically compared to their respective controls (** *p* < 0.01; *n* = 3 blots).

**Figure 12:**
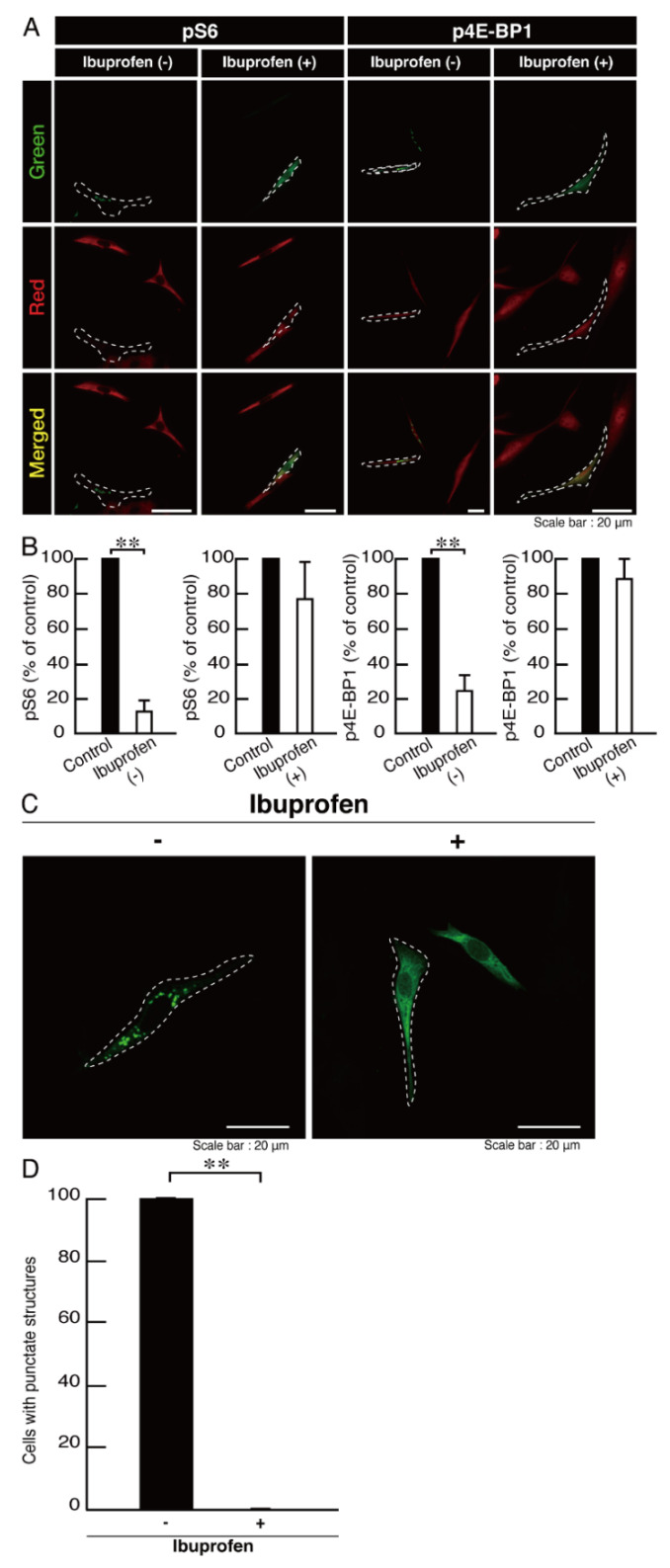
Ibuprofen improves phosphorylation levels of ribosomal S6 and 4E-BP1 proteins and aggregate-like punctate structures in cells harboring the mutants. (**A**,**B**) Cells harboring POLR3B (R550X) (green) in the presence or absence of ibuprofen were stained with an anti-(pS240 and pS244)ribosomal S6 protein (pS6) or anti-(pT37)4E-BP1 (p4E-BP1) antibody (red). Their phosphorylation levels are shown statistically compared to those in their respective control cells under each image (** *p* < 0.01; *n* = 3 fields). (**C**,**D**) Cells harboring POLR3B (R550X) (green) were treated with or without ibuprofen. Percentages of cells with punctate structures were statistically assessed (** *p* < 0.01; *n* = 3 fields).

Ibuprofen is an activator of mTOR signaling [31,32,33,34], and we investigated whether ibuprofen improves the phenotypes of FBD-102b cells harboring the R550X mutant constructs of POLR3B. Treatment with ibuprofen recovered an inhibition of morphological differentiation in cells harboring the mutated constructs (Figure 11A,B), which is consistent with the increasing expression levels of differentiation and myelin marker proteins (Figure 11C,D). Additionally, ibuprofen recovered the decreased ribosomal S6 and 4E-BP1 protein phosphorylation levels (Figure 12A,B). We also explored the effects of ibuprofen on POLR3B mutated protein aggregation. Treatment with ibuprofen recovered the mutated protein aggregation (Figure 12C,D).

**Figure 13:**
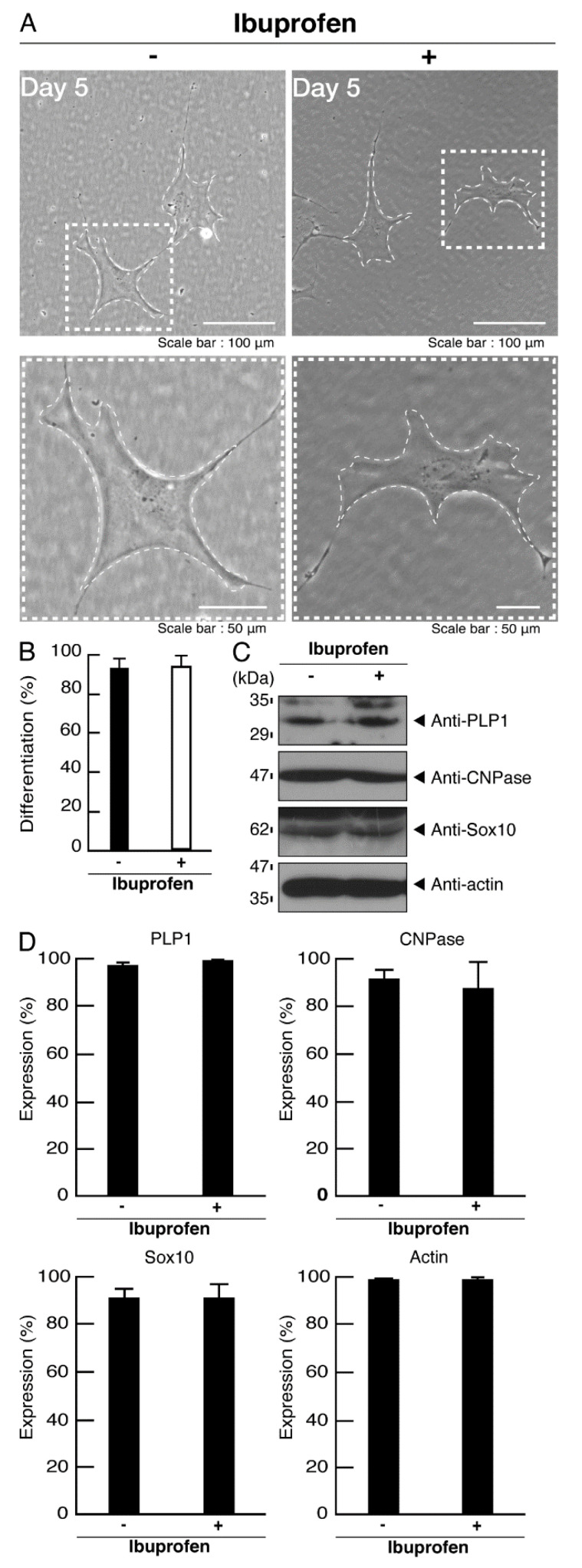
Ibuprofen does not have significant effects on phenotypes in cells harboring the wild type POLR3B. (**A**) Cells harboring the wild type were allowed to differentiate in the presence or absence of ibuprofen for 5 days. Some cells in the upper images are surrounded by white dotted lines. The square fields indicated by the dotted lines in the upper panels are magnified in the respective lower panels. (**B**) Cells with widespread membranes were statistically assessed (*n* = 5 fields (50 cells in total)). (**C**) The lysates of the respective cells were immunoblotted with an antibody against PLP1, CNPase, Sox10, and actin. (**D**) Their expression levels are shown statistically compared to their respective controls (*n* = 3 blots).

**Figure 14:**
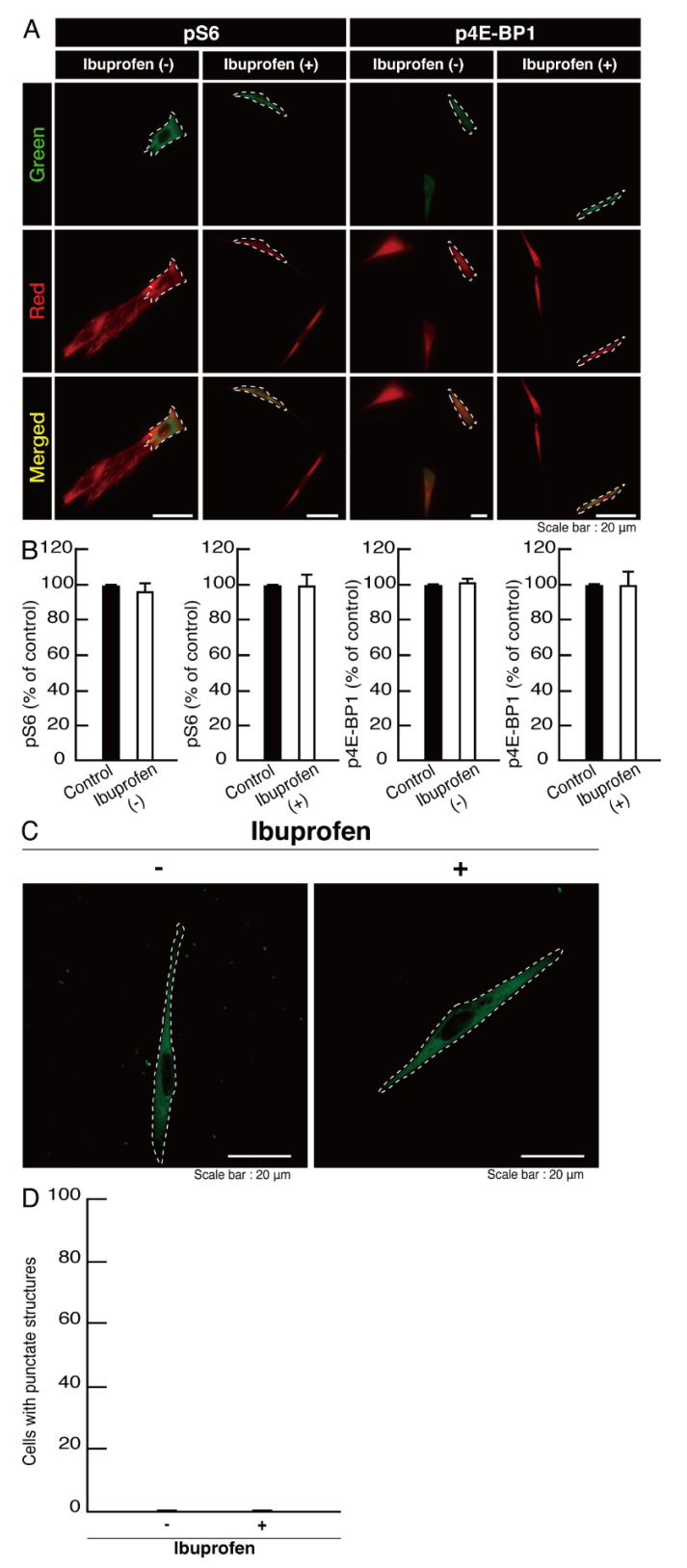
Ibuprofen does not have significant effects on phosphorylation levels of ribosomal S6 and 4E-BP1 proteins and aggregate-like punctate structures in cells harboring the wild type POLR3B. (**A**,**B**) Cells harboring the wild type (green) in the presence or absence of ibuprofen were stained with an anti-(pS240 and pS244)ribosomal S6 protein (pS6) or anti-(pT37)4E-BP1 (p4E-BP1) antibody (red). Their phosphorylation levels are shown statistically compared to those in their respective control cells under each image (*n* = 3 fields). (**C**,**D**) Cells harboring the wild type (green) were treated with or without ibuprofen. The percentage of cells with punctate structures were statistically assessed (*n* = 3 fields).

However, when cells expressing wild type proteins were treated with ibuprofen, ibuprofen had no significant effects on cell morphological differentiation (Figure 13A,B). These effects were supported by the comparable differentiation and myelin marker protein expression levels (Figure 13C,D). Additionally, ibuprofen neither affected phosphorylation levels of ribosomal S6 and 4E-BP1 proteins (Figure 14A,B) nor caused aggregation (Figure 14C,D).

Taken together with the results in cells expressing the wild type proteins, ibuprofen specifically recovers protein aggregation and inhibitory morphological differentiation as well as downregulation of the related signaling in cells expressing the R550X mutant proteins of POLR3B.

## 4. Discussion

Here, we showed that HLD8-associated POLR3B nonsense mutant proteins are localized as aggregates in lysosomes. However, the wild type proteins are not localized in lysosomes. These findings are supported by the results that the mutated but not the wild type proteins were co-localized with a LAMP1 antigen but not KDEL and GM130 antigens. Additionally, in non-denaturing polyacrylamide gel electrophoresis, mutated proteins exhibit a high molecular weight, whereas wild type proteins correspond to the predicted molecular weight. In denaturing polyacrylamide gel electrophoresis, the mutated and wild type proteins correspond to the predicted molecular weight. We also found that cells expressing the mutated proteins show a decreased ability to differentiate morphologically and decreased phosphorylation/activation levels of molecules associated with mTOR signaling; conversely, cells expressing wild type proteins retain their normal abilities. These phenotypes were consistent with changes in differentiation marker protein expression levels. Inhibition of differentiation by the mutated proteins might be consistent with the fact that HLD8 patients show hypomyelination in various brain regions [2,3,4]. Finally, we have identified ibuprofen as a drug that recovers the decreased differentiating ability in cells expressing the mutated proteins. Ibuprofen likely has glial cell-protective effects and the ability to promote cell morphological differentiation. Despite increasing identification of HLD-responsible genes, the molecular mechanisms underlying HLDs have remained unclear. Additionally, the therapeutic procedure has not been established. This is the first report that shows how HLD8-associated POLR3B nonsense mutant proteins inhibit oligodendroglial cell morphological differentiation and how ibuprofen recovers HLD8-associated defective morphological differentiation.

R550X mutation results in a premature stop of POLR3B protein synthesis, which generates proteins with deficient functional domains, and this mutation is thought be a loss-of-function mutation. In this study, the R550X mutation was suggested to cause protein aggregation and its accumulation in lysosomes. This accumulation probably affects signals around lysosomes and morphological differentiation, and on the basis of these results, the R550X mutation can correspond to a gain of function. Although HLD8 includes some other POLR3B mutations, the respective mutations may increase protein aggregation and/or may display a functional deficiency in POLR3B. Thus, possible molecular and cellular pathological effects of mutations in POLR3B may be similarly observed for HLD3-associated mutation of aminoacyl tRNA synthetase complex interacting multifunctional protein 1 (AIMP1) [26], HLD15-associated mutation of glutamyl-prolyl-tRNA synthetase 1 (EPRS1) [17], and HLD17-associated mutation of AIMP2 [18].

It remains unclear which ibuprofen target molecule is involved in promoting morphological differentiation. In contrast to an unanticipated involvement of ibuprofen in oligodendroglial cells in this study, ibuprofen is known to have multiple effects on neuronal cells and tissues. Whether direct or indirect, many ibuprofen target molecules and pathways with protective effects have been described besides cyclooxygenases, such as Cox1/2 [25], which show antipyretic effects (Table 2). Among them, one target is amyloid precursor protein (APP) or amyloid oligomers such as an APP pathological product peptide or other protein oligomers [35,36,37,38]. Ibuprofen inhibits APP secretion and binds pathological amyloid to decrease its toxicity. These reactions are direct effects of ibuprofen. It tightly binds to protein structures such as pathological amyloid proteins to decrease their structural toxicity. Because treating cells with ibuprofen decreases mutated POLR3B protein aggregation, it is possible that ibuprofen can eliminate protein aggregation by binding to the mutated proteins. However, it remains unclear whether the mutated POLR3B proteins display a beta-amyloid-like protein structure.

Another ibuprofen target in neuronal cells is the inhibition of molecule(s) that may be associated with Rho signaling [39,40,41,42,43]. RhoA and related small GTPases as well as Rho-kinases of their effectors are negative regulators of axonal process elongation, whereas Rac1 and Cdc42, which are Rho-family small GTPase members, act as positive regulators of neuronal process elongation. The inhibition of Rho signaling in vitro or in vivo leads to a reverse of neuronal damage following pathological situations such as spinal cord injury. Because oligodendroglial cells also grow multiple processes during their morphological differentiation [9,10,11,12], it is conceivable that ibuprofen participates in oligodendroglial process elongation. Considering all the findings concerning potential ibuprofen targets, ibuprofen may act on all intracellular target molecules at the same or at different times, probably to protect cells and to promote cell differentiation with or without decreasing protein aggregation. No matter which molecular mechanisms underlie treatment with ibuprofen, the results of this study may lead to new disease applications for ibuprofen such as hypomyelinating or demyelinating disease. Ibuprofen may be added to the list of potential HLD8 or other HLD therapeutic drugs.

POLR3B is a key subunit that constitutes RNA polymerase III [1,2,3,4]. Because RNA polymerase III plays a key role in synthesizing essential non-coding RNAs, such as tRNAs and rRNAs, functional defects in POLR3B are thought to have serious effects on normal tissue and organ development. The defects are typically associated with hypodontia and hypogonadotropic hypogonadism, and specially with brain hypomyelination. However, it is unclear why POLR3B abnormalities have particularly prominent and serious effects on oligodendroglial cells. It might be because during development, oligodendroglial cells need a large amount of tRNAs and rRNAs to undergo dynamic morphological changes. Alternatively, POLR3B may have an unidentified oligodendroglial cell-specific particular role. For example, POLR3B may participate in producing RNAs, such as tRNAs and rRNAs, that have high levels of or that are preferentially specific for oligodendroglial cells [44,45,46,47]. Furthermore, POLR3B may have a dual function, similar to AIMP1, which functions as an adaptor protein of amino acid-tRNA ligases and has a role as a precursor for a cytokine called endothelial monocyte-activating polypeptide II (EMAPII) [48]. If this is the case, one of POLR3B’s identified or unidentified roles may be specific for oligodendroglial cells.

Here, we have demonstrated that the HLD8-associated mutation of POLR3B causes POLR3B proteins to aggregate in lysosomes, which decreases the related signaling. The decreased signaling is probably related to the inhibition of oligodendroglial cell morphological differentiation. Ibuprofen recovers the POLR3B mutated protein-mediated inhibition of morphological differentiation and protein aggregation. Similar observations were obtained in studies with HLD7 [49]. Further studies will allow us to understand the detailed molecular mechanisms of how the HLD8-associated mutation of POLR3B inhibits morphological differentiation and also how ibuprofen recovers these possible molecular and cellular pathological effects. Additionally, it can be an important issue to determine whether ibuprofen has effects on HLD8 model mice as well as whether molecular and cellular mechanistic phenotypes seen in cells are preserved in HLD8 model mice. Such studies may lead to the development of the target-specific medications for HLD8 and possibly other HLDs.

## 5. Limitation of This Study and Perspective

The molecular mechanisms and amelioration underlying HLD8 have been clarified at molecular and cellular levels in this study. However, defective morphological differentiation remains unclear, specifically the relationship between no widespread membrane phenotypes and hypomyelinating phenotypes. Future research directions may include clarifying these relationships using disease-specific human induced pluripotent stem cells (iPSCs) or human induced oligodendroglial cells (iOCs). Studies will also lead to experiments in human or mouse oligodendroglial cell–neuronal cocultures and/or crossbreeding of disease model mice with genetically modified animals. In the future, ibuprofen might be used with caution in HLD8 patients because it has many applications in humans. Further studies will clarify if known drug(s) can be used for new applications using a model in this study or the improved models in mice and humans.

## Figures and Tables

**Table 1:** Antibodies used in these experiments.

Product Name	Target or Description	Company	Cat. No.	Lot. No.	Final Conc.
Anti-eIF4EBP1 (phospho T37) antibody	A phospho-peptide corresponding to residues surrounding threonine 37 of eIF4EBP1	abcam	ab75767	GR88680-14	IF, 1/100; IB, 1/2500
Anti-F-actin antibody	Filamentous actin (F-actin)	abcam	ab205	GR319251-7	IB, 1/80,000
Anti-GFP mAb	Green fluorescent protein (GFP)	MBL	598	“078”	IB, 1/80,000
Anti-KDEL mAb	KDEL-containing peptide of the endoplasmic reticulum (ER)-resident glucose-regulated protein (GRP78)	MBL	M181-3	“004”	IF, 1/500
Anti-PLP1 antibody	Myelin proteolipid protein (PLP) 1	Atlas Antibodies	HPA004128	B115828	IB, 1/500
Anti-RPS6 (phospho S240 + S244) antibody	Synthetic peptide within human S6 protein to the C-terminus (phospho S240 + S244)	abcam	ab215214	GR3205097-3	IF, 1/101; IB, 1/10,000
CNPase (D83E10) XP Rabbit mAb	2′, 3′-cyclic nucleotide 3′-phospho-diesterase (CNPase)	Cell Signaling TECHNOLOGY	#5664	“001”	IB, 1/500
Anti-LAMP-1 antibody (H4A3)	Lysosomal-associated membrane protein1 (LAMP1)	Santa Cruz Biotechnology	sc-20011	J0919	IF, 1/200
Purified Mouse Anti-GM130 antibody	Golgi matrix protein of 130 kDa (GM130)	BD Biosciences	610823	8352796	IF, 1/501
Anti-Sox-10 antibody (A-2)	SRY-related HMG-box 10 (Sox-10)	Santa Cruz Biotechnology	sc-365692	J0720	IB, 1/500
Anti-elF4EBP1 (Y329)	elF4EBP1	abcam	ab32024	GR239794-12	IB, 1/5000
Ribosomal Protein S6 (C-8)	Ribosomal Protein S6	Santa Cruz Biotechnology	sc-74459	D2921	IB, 1/500
Alexa Fluor 488 goat anti-mouse IgG (H + L)	Mouse IgG (H + L) conjugated with Alexa Fluor 488	Thermo Fisher Scientific	A-11001	774904	IF, 1/500
Alexa Fluor 488 goat anti-rabbit IgG (H + L)	Rabbit IgG (H + L) conjugated with Alexa Fluor 488	Thermo Fisher Scientific	A-11008	751094	IF, 1/500
Alexa Fluor 594 goat anti-mouse IgG (H + L)	Mouse IgG (H + L) conjugated with Alexa Fluor 594	Thermo Fisher Scientific	A-11005	2043369	IF, 1/500
Alexa Fluor 594 goat anti-rabbit IgG (H + L)	Rabbit IgG (H + L) conjugated with Alexa Fluor 594	Thermo Fisher Scientific	A-11012	2018240	IF, 1/500
Anti-IgG (H + L chain) (Mouse) pAb-HRP	Mouse IgG F(ab’) conjugated with horseradish peroxidase	MBL	330	366	IB, 1/5000
Anti-IgG (H + L chain) (Rabbit) pAb-HRP	Rabbit IgG F(ab’) conjugated with horseradish peroxidase	MBL	458	352	IB, 1/5000

**Table 2:** Molecular targets of ibuprofen and its effects on cells and tissues. Up arrow indicates upregulation or increase whereas down arrow shows downregulation or decrease.

Molecular Target and/or Mechanism	Animal or Human Model	Disease	Effect	Reference
Alpha-synuclein oligomers (alpha-synOs)	Mouse model (in vivo)	Synucleinopathies	memory impairment induced by alpha-synOs (↓)	Pietro La Vitola, Claudia Balducci, Milica Cerovic, Giulia Santamaria, Edoardo Brandi, Federica Grandi, Laura Caldinelli, Laura Colombo, Maria Grazia Morgese, Luigia Trabace, Loredano Pollegioni, Diego Albani, Gianluigi Forloni: Alpha-synuclein oligomers impair memory through glial cell activation and via Toll-like receptor 2. Brain, Behavior, and Immunity 2018 69:591–602
Amyloid-beta peptide (Abeta)	Rat model (in vitro)	Alzheimer’s disease (AD)	alter the ultrastructure of Abeta, its association to neuronal membranes and its synaptotoxic effect (↓)	Maria Paz Zurita, Gonzalo Muñoz, Fernando J Sepúlveda, Paulina Gómez, Carolina Castillo, Carlos F Burgos, Jorge Fuentealba, Carlos Opazo, Luis G Aguayo: Ibuprofen inhibits the synaptic failure induced by the amyloid-β peptide in hippocampal neurons. Journal of Alzheimer’s Disease 2013 35:463–473
Abeta	Human model (in vitro)		may alter the ultrastructure of Abeta	Mohammad Khursheed Siddiqi, Parvez Alam, Sadia Malik, Nabeela Majid, Sumit Kumar Chaturvedi, Sudeepa Rajan, Mohd Rehan Ajmal, Mohsin Vahid Khan, Vladimir N Uversky, Rizwan Hasan Khan: Stabilizing proteins to prevent conformational changes required for amyloid fibril formation. Journal of Cellular Biochemistry 2019 12:2642–2656
Abeta	Rat model (in vitro)		may alter the ultrastructure of Abeta	Tanja Hochstrasser, Lindsay A Hohsfield, Barbara Sperner-Unterweger, Christian Humpel: Beta-Amyloid-induced effects on cholinergic, serotonergic, and dopaminergic neurons are differentially counteracted by anti-inflammatory drugs. Journal of Neuroscience Research 2013 91:83–94
Apolipoprotein (APO) E4	Mouse model (in vivo)	AD	alter the expression pattern of APOE in APOE4 mice to that of APOE3 mice, dendritic spine density (↑)	Amanda M DiBattista, Sonya B Dumanis, Joshua Newman, G William Rebeck: Identification and modification of amyloid-independent phenotypes of APOE4 mice. Experimental Neurology 2016 280:97–105
beta amyloid precursor protein (betaAPP)	Human model (in vitro)	AD	the secretion of total Abeta (↓), the accumulation of Abeta-42 and Abeta-40 (↓)	I Blasko, A Apochal, G Boeck, T Hartmann, B Grubeck-Loebenstein, G Ransmayr: Ibuprofen decreases cytokine-induced amyloid beta production in neuronal cells. Neurobiology of Disease 2001 8:1094–1101
Beta-site amyloid precursor protein cleaving enzyme1 (BACE1), advanced glycation end product (AGE) and receptor for AGE (RAGE)	Rat model (in vivo)	Diabetic encephalopathy	the decline in learning and memory ability and loss of neurons in the CA1 and CA3 areas of the hippocampus (↓)	Yao-Wu Liu, Xia Zhu, Liang Zhang, Qian Lu, Fan Zhang, Hao Guo, Xiao-Xing Yin: Cerebroprotective effects of ibuprofen on diabetic encephalopathy in rats. Pharmacology Biochemistry and Behavior 2014 117:128–136
Cyclooxygenase (Cox) family	Rat model (in vivo)	Forebrain-ischemia	post-ischemic thromboxane B2 and prostaglandin F2alpha in both caudate nucleus and dorsal hippocampus and neuronal injury (↓)	P M Patel, J C Drummond, T Sano, D J Cole, C J Kalkman, T L Yaksh: Effect of ibuprofen on regional eicosanoid production and neuronal injury after forebrain ischemia in rats. Brain Research 1993 614:315–324
Cox1/2	Rat model (in vitro)	Parkinson’s disease (PD)	1-methyl-4-phenylpyridinium (MPP(+))-induced VM cell toxicity and VM dopaminergic cell apoptosis (↓)	Ya-Ching Hsieh, Ross B Mounsey, Peter Teismann: MPP(+)-induced toxicity in the presence of dopamine is mediated by COX-2 through oxidative stress. Naunyn-Schmiedeberg’s Archives of Pharmacolog 2011 384:157–167
Cox1/2	Mouse model (in vivo)	PD	protect neurons against 1-methyl-4-phenyl-1,2,3,6-tetrahydropyridine (MPTP)-induced injury in the striatum	Maciej Swiątkiewicz, Małgorzata Zaremba, Ilona Joniec, Andrzej Członkowski, Iwona Kurkowska-Jastrzębska: Potential neuroprotective effect of ibuprofen, insights from the mice model of Parkinson’s disease. Pharmacological Reports 2013 65:1227–1236
Cox1/2	Mouse model (in vivo)	AD	modulate hippocampal gene expression in pathways involved in neuronal plasticity and norepinephrine and dopamine (↑)	Nathaniel S Woodling, Damien Colas, Qian Wang, Paras Minhas, Maharshi Panchal, Xibin Liang, Siddhita D Mhatre, Holden Brown, Novie Ko, Irene Zagol-Ikapitte, Marieke van der Hart, Taline V Khroyan, Bayarsaikhan Chuluun, Prachi G Priyam, Ginger L Milne, Arash Rassoulpour, Olivier Boutaud, Amy B Manning-Boğ, H Craig Heller, Katrin I Andreasson: Cyclooxygenase inhibition targets neurons to prevent early behavioural decline in Alzheimer’s disease model mice. Brain 2016 139:2063–2081
Cox2	Rat model (in vivo)	Stroke	impairment of experience-dependent plasticity (↓), the Cox2 after stroke (↓)	Jan A Jablonka, Malgorzata Kossut, Otto W Witte, Monika Liguz-Lecznar: Experience-dependent brain plasticity after stroke: effect of ibuprofen and poststroke delay. European Journal of Neuroscience 2012 36:2632–2639
Cox2	Mouse model (in vivo)	Psychiatric disorders and neurodegenerative diseases	anti-inflammatory markers (↑), tryptophan 2,3-dioxygenase (TDO2) and PGE2 (↓)	Maria Teresa Golia, Silvia Poggini, Silvia Alboni, Stefano Garofalo, Naomi Ciano Albanese, Aurelia Viglione, Maria Antonietta Ajmone-Cat, Abygaël St-Pierre, Nicoletta Brunello, Cristina Limatola, Igor Branchi, Laura Maggi: Interplay between inflammation and neural plasticity: Both immune activation and suppression impair LTP and BDNF expression. Brain, Behavior, and Immunity 2019 81:484–494
Cox2 and nucleotide-binding oligomerization domain (NOD) -like receptor 3 inflammasome	Rat model (in vivo)	Pentylenetetrazol-induced chronic epilepsy	seizure scores and the secretion of the inflammatory cytokine interleukin (IL)-18 (↓), loss of hippocampal neurons (↓)	Rui Liu, Shuhua Wu, Chong Guo, Zhongbo Hu, Jiangtao Peng, Ke Guo, Xinfan Zhang, Jianmin Li: Ibuprofen Exerts Antiepileptic and Neuroprotective Effects in the Rat Model of Pentylenetetrazol-Induced Epilepsy via the COX-2/NLRP3/IL-18 Pathway. Neurochemical Research 2020 45:2516–2526
Cox2 and PGJ2	Rat model (in vivo)	PD	dopaminergic neuronal loss (↓)and amoeboid microglia in the substantia nigra (↓), and the bias toward use of the ipsilateral over the contralateral forelimb (↓)	Chuhyon Corwin, Anastasia Nikolopoulou, Allen L Pan, Mariela Nunez-Santos, Shankar Vallabhajosula, Peter Serrano, John Babich, Maria E Figueiredo-Pereira: Prostaglandin D2/J2 signaling pathway in a rat model of neuroinflammation displaying progressive parkinsonian-like pathology: potential novel therapeutic targets. Journal of Neuroinflammation 2018 15:272
Cox1/2 and arachidonic acid cascade	Mouse and rat models (in vitro, in vivo)	PD	the neuroinflammatory response (↓)	Ashish Singh, Pratibha Tripathi, Sarika Singh: Neuroinflammatory responses in Parkinson’s disease: relevance of Ibuprofen in therapeutics. Inflammopharmacology 2021 29:5–14
Inducible nitric oxide synthase (iNOS)	Rat model (in vitro)	AD	iNOS mRNA and protein (↓)	N C Stratman, D B Carter, V H Sethy: Ibuprofen: effect on inducible nitric oxide synthase. Molecular Brain Research 1997 50:107–112
IL-1 receptor antagonist proteins	Rat model (in vitro, in vivo)	Cerebral ischemia	protect CA1 hippocampal neurons for a long time, neurons (↑)	E-M Park, B-P Cho, B T Volpe, M O Cruz, T H Joh, S Cho: Ibuprofen protects ischemia-induced neuronal injury via upregulating interleukin-1 receptor antagonist expression. Neuroscience 2005 132:625–631
Microglial nicotinamide adenine dinucleotide phosphate (NADPH) oxidase (NOX2)	Human model (in vitro), mouse model (in vivo)	AD	oxidative damage (↓) and plaque clearance in the brain (↑)	Brandy L Wilkinson, Paige E Cramer, Nicholas H Varvel, Erin Reed-Geaghan, Qingguang Jiang, Alison Szabo, Karl Herrup, Bruce T Lamb, Gary E Landreth: Ibuprofen attenuates oxidative damage through NOX2 inhibition in Alzheimer’s disease. Neurobiology of Aging 2012 33:197.e21–e32
Mitogen-activated protein kinases (MAPKs) etc.	Rat model (in vivo)	PD and cypermethrin-induced Parkinsonism	cypermethrin-induced pathophysiological effects along with expression of pro-inflammatory and/or apoptosis-related proteins in the nigrostriatal tissue (↓)	Ashish Singh, Pratibha Tripathi, Om Prakash, Mahendra Pratap Singh: Ibuprofen abates cypermethrin-induced expression of pro-inflammatory mediators and mitogen-activated protein kinases and averts the nigrostriatal dopaminergic neurodegeneration. Molecular Neurobiology 2016 53:6849–6858
Mitogen-activated protein kinases (MAPKs) etc.	Rat model (in vivo)	cypermethrin-induced Parkinsonism	may resist cypermethrin-induced pathophysiological effects	Pratibha Tripathi, Ashish Singh, Lakshmi Bala, Devendra Kumar Patel, Mahendra Pratap Singh: Ibuprofen Protects from Cypermethrin-Induced Changes in the Striatal Dendritic Length and Spine Density. Molecular Neurobiology 2018 55:2333–2339
Neuroglobin	Rat model (in vivo)	AD	neuroglobin (↑), protein kinase B (Akt) signaling (↑)	Susi Zara, Marianna De Colli, Monica Rapino, Stephanie Pacella, Cinzia Nasuti, Piera Sozio, Antonio Di Stefano, Amelia Cataldi: Ibuprofen and lipoic acid conjugate neuroprotective activity is mediated by Ngb/Akt intracellular signaling pathway in Alzheimer’s disease rat model. Gerontology 2013 59:250–260
Neuroinflammatory mediator molecules	Rat model (in vivo)	Hypoxia-ischemia	the P3 HI-induced reductions in brain serotonin levels, serotonin transporter expression, and numbers of serotonergic neurons in the dorsal raphé nuclei (↓)	Julie A Wixey, Hanna E Reinebrant, Kathryn M Buller: Post-insult ibuprofen treatment attenuates damage to the serotonergic system after hypoxia-ischemia in the immature rat brain. Journal of Neuropathology Experimental Neurology 2012 71:1137–1148
Neuronal pentraxin	Mouse model (in vivo)	AD	cognitive function (↑), IL-1beta (↓)	Anum Jamil, Aamra Mahboob, Touqeer Ahmed: Ibuprofen targets neuronal pentraxins expression and improves cognitive function in mouse model of AlCl 3-induced neurotoxicity. Experimental and Therapeutic Medicine 2016 11:601–606
Nuclear factor (NF) kappa B inhibitor alpha protein and dopamine- and cAMP-regulated phosphoprotein-32 (DARPP-32) neuronal marker	Human model (in vitro), mouse model (in vivo)	Machado–Joseph disease	synaptic function and neural progenitors proliferation markers (↑), neuropathology and motor coordination (↑)	Liliana S Mendonça, Clévio Nóbrega, Silvia Tavino, Maximilian Brinkhaus, Carlos Matos, Sandra Tomé, Ricardo Moreira, Daniel Henriques, Brian K Kaspar, Luís Pereira de Almeida: Ibuprofen enhances synaptic function and neural progenitors proliferation markers and improves neuropathology and motor coordination in Machado–Joseph disease models. Human Molecular Genetics 2019 28:3691–3703
Protein kinase C epsilon-mediated matrix metalloproteinase-2/9 (PKC epsilon-mediated MMP-2/9)	Rat model (in vivo)	AD	PKC epsilon-mediated MMP-2 and MMP-9 (↓), control symptoms of AD	S Zara, M Rapino, P Sozio, A Di Stefano, C Nasuti, A Cataldi: Ibuprofen and lipoic acid codrug 1 control Alzheimer’s disease progression by downregulating protein kinase C ε-mediated metalloproteinase 2 and 9 levels in β-amyloid infused Alzheimer’s disease rat model. Brain Research 2011 1412:79–87
RhoA	Human and chick models (in vitro), mouse and rat models (in vitro)	Spinal cord injury	ligand-induced Rho signaling and myelin-induced inhibition (↓), the recovery of rats from a clinically relevant spinal cord trauma (↑)	Xingxing Wang, Stephane Budel, Kenneth Baughman, Grahame Gould, Kang-Ho Song, Stephen M Strittmatter: Ibuprofen enhances recovery from spinal cord injury by limiting tissue loss and stimulating axonal growth. Journal of Neurotrauma 2009 26:81–95
RhoA	Rat model (in vitro, in vivo)	Spinal cord injury	may protect spinal cord injury	Bin Xing, Hui Li, Hongyu Wang, Dhriti Mukhopadhyay, Daniel Fisher, Christopher J Gilpin, Shuxin Li: RhoA-inhibiting NSAIDs promote axonal myelination after spinal cord injury. Experimental Neurology 2011 231:247–260
RhoA	Human model (in vitro)	Axonal injury	may protect axonal injury	Frank Roloff, Hannah Scheiblich, Carola Dewitz, Silke Dempewolf, Michael Stern, Gerd Bicker: Enhanced neurite outgrowth of human model (NT2) neurons by small-molecule inhibitors of Rho/ROCK signaling. PLoS One 2015 10:e0118536
RhoA	Human and mouse models (in vitro)	Neurodegenerative diseases	RhoA activation and microglial phagocytosis of neuronal cell fragments (↓)	Hannah Scheiblich, Gerd Bicker: Regulation of Microglial Phagocytosis by RhoA/ROCK-Inhibiting Drugs. Cellular and Molecular Neurobiology 2017 37:461–473
RhoA	Human model (in vitro)	AD	neurite collapse and formation of stress fibers induced by Abeta (↓)	Patricia Ferrera, Angélica Zepeda, Clorinda Arias: Nonsteroidal anti-inflammatory drugs attenuate amyloid-β protein-induced actin cytoskeletal reorganization through Rho signaling modulation. Cellular and Molecular Neurobiology 2017 37:1311–1318
Transcription factor peroxisome proliferator-activated receptor gamma (PPARgamma)	Rat model (in vitro)	AD and spinal cord injury	amyloid-beta42 peptide (↓) via inactivation of RhoA signaling	John Dill, Ankur R Patel, Xiao-Li Yang, Robert Bachoo, Craig M Powell, Shuxin Li: A molecular mechanism for ibuprofen-mediated RhoA inhibition in neurons. The Journal of Neuroscience 2010 30:963–972
Unknown	Mouse model (in vitro, in vivo)	AD	plaque burden (↓)	Ji-Kyung Choi, Bruce G Jenkins, Isabel Carreras, Sukru Kaymakcalan, Kerry Cormier, Neil W Kowall, Alpaslan Dedeoglu: Anti-inflammatory treatment in AD mice protects against neuronal pathology. Experimental Neurology 2010 223:377–384
Unknown	Mouse model (in vivo)	AD	neuritic plaque pathology and inflammation (↓)	Dikmen Dokmeci: Ibuprofen and Alzheimer’s disease. Folia Med 2004 46:5–10
Unknown	Mouse model (in vitro, in vivo)	Ataxia telangiectasia	cytodegeneration, cytological damage, young mutant pups’ LPS-induced behavioral deficits (↓)	Chin Wai Hui, Xuan Song, Fulin Ma, Xuting Shen, Karl Herrup: Ibuprofen prevents progression of ataxia telangiectasia symptoms in ATM-deficient mice. Journal of Neuroinflammation 2018 15:308
Unknown	Piglet model (in vivo)	Intrauterine growth restriction	the inflammatory response and neuronal and matter impairment (↓)	Julie A. Wixey, Kishen R. Sukumar, Rinaldi Pretorius, Kah Meng Lee, Paul B. Colditz, S. Tracey Bjorkman, Kirat K. Chand: Ibuprofen Treatment Reduces the Neuroinflammatory Response and Associated Neuronal and White Matter Impairment in the Growth Restricted Newborn. Frontiers in Physiology 2019 10:541
Unknown	Mouse model (in vitro)	Cerebral ischemia	neuronal cell death induced by kainate excitotoxicity or N-methyl-D-aspartate (↓)	Yusuke Iwata, Olivier Nicole, David Zurakowski, Toru Okamura, Richard A Jonas: Ibuprofen for neuroprotection after cerebral ischemia. The Journal of Thoracic and Cardiovascular Surgery 2010 139:489–493
Unknown	Rat model (in vivo)	PD	reverse rotenone-induced motor deficits and depressive-like behavior	Tiago Zaminelli, Raísa Wendhausen Gradowski, Taysa Bervian Bassani, Janaína Kohl Barbiero, Ronise M Santiago, Daniele Maria-Ferreira, Cristiane Hatsuko Baggio, Maria A B F Vital: Antidepressant and antioxidative effect of Ibuprofen in the rotenone model of Parkinson’s disease. Neurotoxicity Research 2014 26:351–362
Unknown	Mouse model (in vivo)	Age-dependent impairment of cognitive function	astrocyte activation (↓), synaptic plasticity and memory function (↑)	Justin T Rogers, Chia-Chen Liu, Na Zhao, Jian Wang, Travis Putzke, Longyu Yang, Mitsuru Shinohara, John D Fryer, Takahisa Kanekiyo, Guojun Bu: Subacute ibuprofen treatment rescues the synaptic and cognitive deficits in advanced-aged mice. Neurobiology of Aging 2017 53:112–121
Unknown	Rat model (in vivo)	Intervertebral foramen inflammation (IVFI)	severity and duration of IVFI-induced thermal hyperalgesia and mechanical allodynia (↓), hyperexcitability of the inflamed DRG neurons (↓)	Zhi-Jiang Huang, Erica Hsu, Hao-Chuan Li, Anthony L Rosner, Ronald L Rupert, Xue-Jun Song: Topical application of compound Ibuprofen suppresses pain by inhibiting sensory neuron hyperexcitability and neuroinflammation in a rat model of intervertebral foramen inflammation. The Journal of Pain 2011 12:141–152
Unknown	Mouse model (in vivo)	Batten disease and juvenile neuronal ceroid lipofuscinosis	the performance on the vertical pole test (concomitant use with lamotrigine) (↑), slightly ameliorate microgliosis	Marta A Tarczyluk-Wells, Christoph Salzlechner, Allison R Najafi, Ming J Lim, David Smith, Frances M Platt, Brenda P Williams, Jonathan D Cooper: Combined Anti-inflammatory and Neuroprotective Treatments Have the Potential to Impact Disease Phenotypes in Cln3−/− Mice. Frontiers in Neurology 2019 10:963
Unknown	Rat model (in vitro)	Glutamate excitotoxicity and PD	dopamine uptake caused by glutamate (↓), protect both dopaminergic neurons and neurons overall against glutamate toxicity	D Casper, U Yaparpalvi, N Rempel, P Werner: Ibuprofen protects dopaminergic neurons against glutamate toxicity in vitro. Neuroscience Letters 2000 289:201–204
Unknown	Mouse model (in vivo)	PD	loss of mesencephalic dopaminergic neurons (↓), the number of CD68+/ Iba-1+ cells, the microglia/neurons interactions, and the pro-inflammatory cytokines (concomitant use with 1-deoxynojirimycin) (↓)	Tcs Costa, E Fernandez-Villalba, V Izura, A M Lucas-Ochoa, N J Menezes-Filho, R C Santana, M D de Oliveira, F M Araújo, C Estrada, Vda Silva, S L Costa, M T Herrero: Combined 1-Deoxynojirimycin and Ibuprofen Treatment Decreases Microglial Activation, Phagocytosis and Dopaminergic Degeneration in MPTP-Treated Mice. Journal of Neuroimmune Pharmacology 2021 16:390–402
Unknown	Rat model (in vivo)	Exercise-induced fatigue	the acetylcholinesterase (AChE) activity (↓), neuronal tumor necrosis factor-alpha (TNF-alpha) and IL-1beta (↓)	F D Lima, D N Stamm, I D Della Pace, L R Ribeiro, L M Rambo, G Bresciani, J Ferreira, M F Rossato, M A Silva, M E Pereira, R P Ineu, A R Santos, F Bobinski, M R Fighera, L F Royes: Ibuprofen intake increases exercise time to exhaustion: A possible role for preventing exercise-induced fatigue. Scandinavian Journal of Medicine & Science in Sports 2016 26:1160–1170
Unknown	Rat model (in vivo)	PD	delay the development of dyskinesia, Cox2 and vascular endothelial growth factor (VEGF) in striatal (↓)	Asmaa M Teema, Sawsan A Zaitone, Yasser M Moustafa: Ibuprofen or piroxicam protects nigral neurons and delays the development of l-dopa induced dyskinesia in rats with experimental Parkinsonism: Influence on angiogenesis. Neuropharmacology 2016 107:432–450
Unknown	Human model (in vitro)	Hypomyelinating leukodystrophy 3	reverse mutant-mediated inhibitory differentiation and the localization in the lysosome.	Yu Takeuchi, Marina Tanaka, Nanako Okura, Yasuyuki Fukui, Ko Noguchi, Yoshihiro Hayashi, Tomohiro Torii, Hiroaki Ooizumi, Katsuya Ohbuchi, Kazushige Mizoguchi, Yuki Miyamoto, Junji Yamauchi: Rare Neurologic Disease-Associated Mutations of AIMP1 are Related with Inhibitory Neuronal Differentiation Which is Reversed by Ibuprofen. Medicines (Basel) 2020 7:25
Unknown	Rodent model (in vivo)	Hypoxia-ischemia	Cox2, IL-1β and TNF-alpha (↓), the loss O4- and O1-positive oligodendrocyte progenitor cells and myelin basic protein (MBP)-positive myelin content (↓)	M L Carty, J A Wixey, H E Reinebrant, G Gobe, P B Colditz, K M Buller: Ibuprofen inhibits neuroinflammation and attenuates white matter damage following hypoxia-ischemia in the immature rodent brain. Brain Research 2011 1402:9–19
Unknown	Mouse model (in vitro, in vivo)	Manganism	the manganese-induced increase in cerebral F(2)-isoprostanes (↓) and protect the MSNs from dendritic atrophy and dendritic spine loss	Dejan Milatovic, Ramesh C Gupta, Yingchun Yu, Snjezana Zaja-Milatovic, Michael Aschner: Protective effects of antioxidants and anti-inflammatory agents against manganese-induced oxidative damage and neuronal injury. Toxicology and Applied Pharmacology 2011 256:219–226
Unknown	Rat model (in vivo)	Intermittent hypoxia	oxidative stress, gp91 (phox) expression and macrophage infiltration in the CB (↓)	Siu-Yin Lam, Yu Liu, Kwong-Man Ng, Chi-Fai Lau, Emily C Liong, George L Tipoe, Man-Lung Fung: Chronic intermittent hypoxia induces local inflammation of the rat carotid body via functional upregulation of proinflammatory cytokine pathways. Histochemistry and Cell Biology 2012 137:303–317
Unknown	Human model (in vitro), mouse model (in vivo)	oxaliplatin(OXA)-induced painful neuropathy	the neurotoxic OXA effects (↓) (a significant dose-dependent decrease in viability, a large increase in reactive oxygen species (ROS) and NO production, lipid peroxidation and mitochondrial impairment)	France Massicot, Guillaume Hache, Ludivine David, Dominique Chen, Charlotte Leuxe, Laure Garnier-Legrand, Patrice Rat, Olivier Laprévote, François Coudoré: P2X7 Cell Death Receptor Activation and Mitochondrial Impairment in Oxaliplatin-Induced Apoptosis and Neuronal Injury: Cellular Mechanisms and In Vivo Approach. PLoS One 2013 8:e66830
Unknown	Human model (in vitro), rat model (in vivo)	Pentylenetetrazol-induced epilepsy	the proliferation of astrocytes (↓) by increasing autophagy	Jiangtao Peng, Shuhua Wu, Chong Guo, Ke Guo, Weiguo Zhang, Rui Liu, Jianmin Li, Zhongbo Hu: Effect of Ibuprofen on Autophagy of Astrocytes During Pentylenetetrazol-Induced Epilepsy and its Significance: An Experimental Study. Neurochemical Research 2019 44:2566–2576
Unknown	Mouse model (in vivo)	Dementia with Lewy bodies	protein aggregation and astrogliosis (↓)	Kazunari Sekiyama, Masayo Fujita, Akio Sekigawa, Yoshiki Takamatsu, Masaaki Waragai, Takato Takenouchi, Shuei Sugama, Makoto Hashimoto: Ibuprofen ameliorates protein aggregation and astrocytic gliosis, but not cognitive dysfunction, in a transgenic mouse expressing dementia with Lewy bodies-linked P123H β-synuclein. Neuroscience Letters 2012 515:97–101
Unknown	Rat model (in vivo)	Traumatic brain injury	CD45 and TGF-beta1 (↓)	T Cao, T C Thomas, J M Ziebell, J R Pauly, J Lifshitz: Morphological and genetic activation of microglia after diffuse traumatic brain injury in the rat. Neuroscience 2012 225:65–75
Unknown	Rat model (in vivo)	Hyperammonemia and hepatic encephalopathy	microglial activation (↓) and cognitive and motor functions (↑)	Regina Rodrigo, Omar Cauli, Ulises Gomez-Pinedo, Ana Agusti, Vicente Hernandez-Rabaza, Jose-Manuel Garcia-Verdugo, Vicente Felipo: Hyperammonemia induces neuroinflammation that contributes to cognitive impairment in rats with hepatic encephalopathy. Gastroenterology 2010 139:675–684
Unknown	Rat model (in vitro)	Hyperprolinemias	the glutamate and glutamine (↓), glutamine synthetase and AChE activities (↓), and acetylcholine (Ach) (↑)	Samanta Oliveira Loureiro, Daniele Susana Volkart Sidegum, Helena Biasibetti, Mery Stefani Leivas Pereira, Diogo Losch de Oliveira, Regina Pessoa-Pureur, Angela T S Wyse: Crosstalk Among Disrupted Glutamatergic and Cholinergic Homeostasis and Inflammatory Response in Mechanisms Elicited by Proline in Astrocytes. Molecular Neurobiology 2016 53:1065–1079

## Data Availability

Not applicable.

## Supplementary Materials

The following are available online at https://www.mdpi.com/article/10.3390/neurolint14010018/s1, Figure S1: Full gel scan images.
