# Supplementary figures and images for: Hypomyelinating Leukodystrophy 8 (HLD8)-Associated Mutation of POLR3B Leads to Defective Oligodendroglial Morphological Differentiation Whose Effect Is Reversed by Ibuprofen
Source: Neurol Int. 2022 Feb 16;14(1):212–44. doi: 10.3390/neurolint14010018 (PMC8884015; doi:10.3390/neurolint14010018)

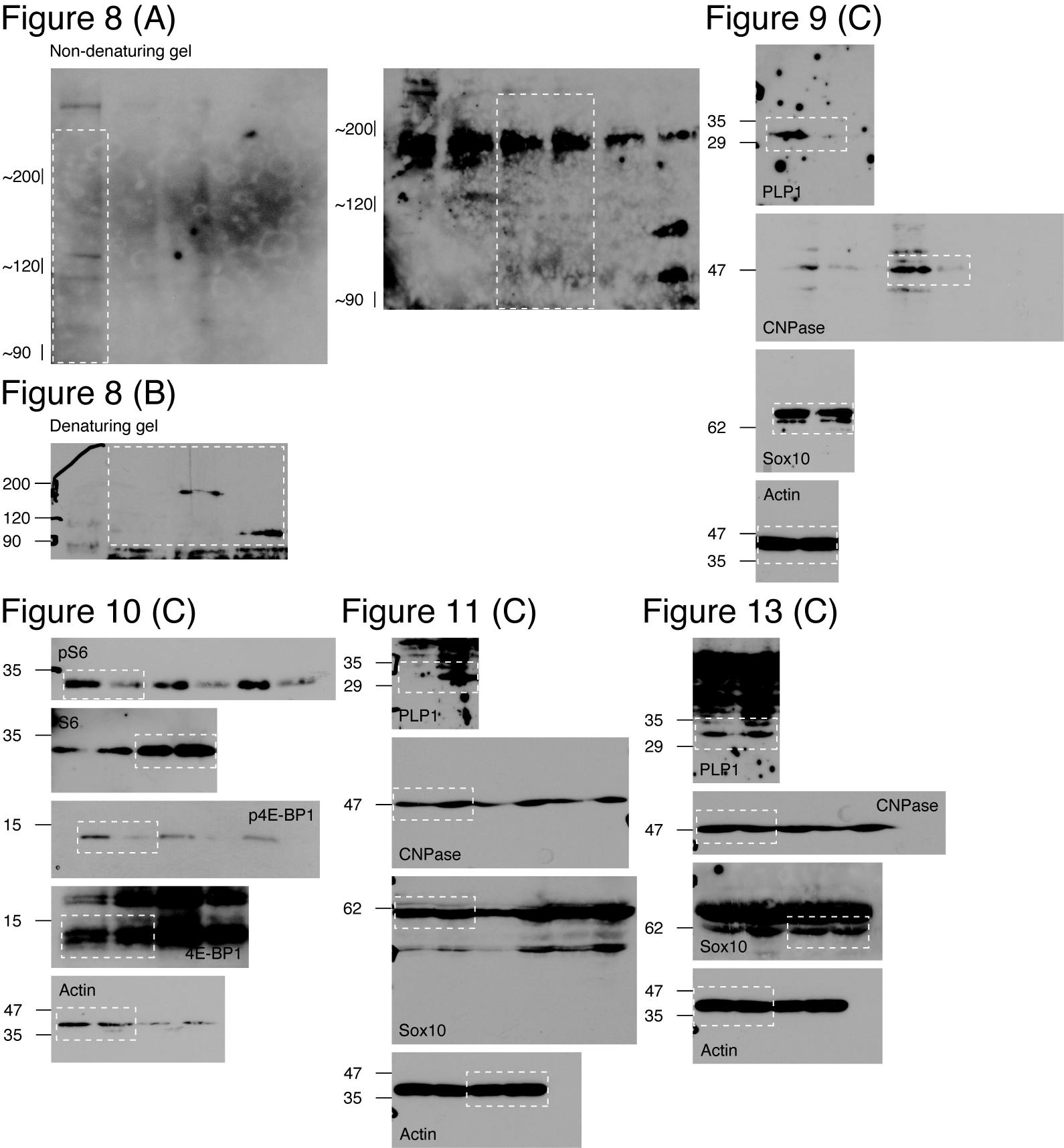

Supplement: Supplementary file 1 [file neurolint-14-00018-s001.zip › neurolint-1478311-supplementary.tif]
